# Supplementary material for: Discovery of RNA-binding proteins and characterization of their dynamic responses by enhanced RNA interactome capture
Source: Nat Commun. 2018 Oct 23;9:4408. doi: 10.1038/s41467-018-06557-8 (PMC6199288; doi:10.1038/s41467-018-06557-8)
Supplement: Supplementary file 1 — Supplementary Information [file 41467_2018_6557_MOESM1_ESM.pdf]

Supplementary Information

Discovery of RNA-binding proteins and characterization of their dynamic responses by enhanced RNA interactome capture

Perez-Perri J.I. *et al.*

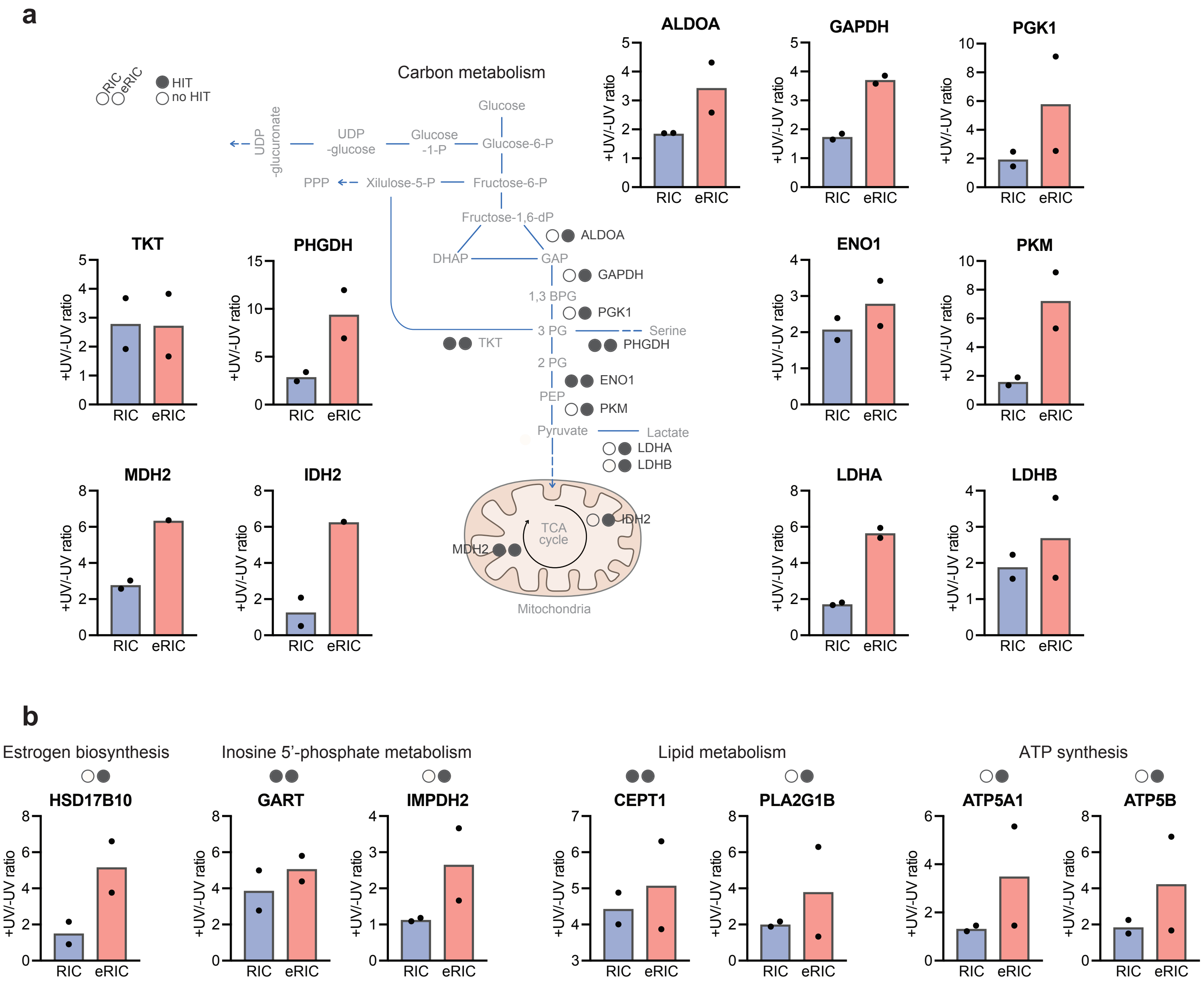

Supplementary Figure 1: eRIC enhances detection of RBPs lacking classical RNA-binding domains. (**a**,**b**) Fold change in irradiated (+UV) over non-irradiated (-UV) samples of enzymes of intermediate metabolism captured by eRIC and RIC. Enzymes of carbon metabolism (**a**) and of other metabolic pathways (**b**) are shown. Note how the enrichment over background for most of these enzymes is enhanced in eRIC samples in relation to RIC. IDH2 and MDH2 were not detected in the -UV control of one eRIC experiment, so the corresponding +UV/-UV ratio is not displayed. Data are shown as mean from two biologically independent experiments. Proteins with FDR < 0.05 (moderated *t*-test) are considered hits.

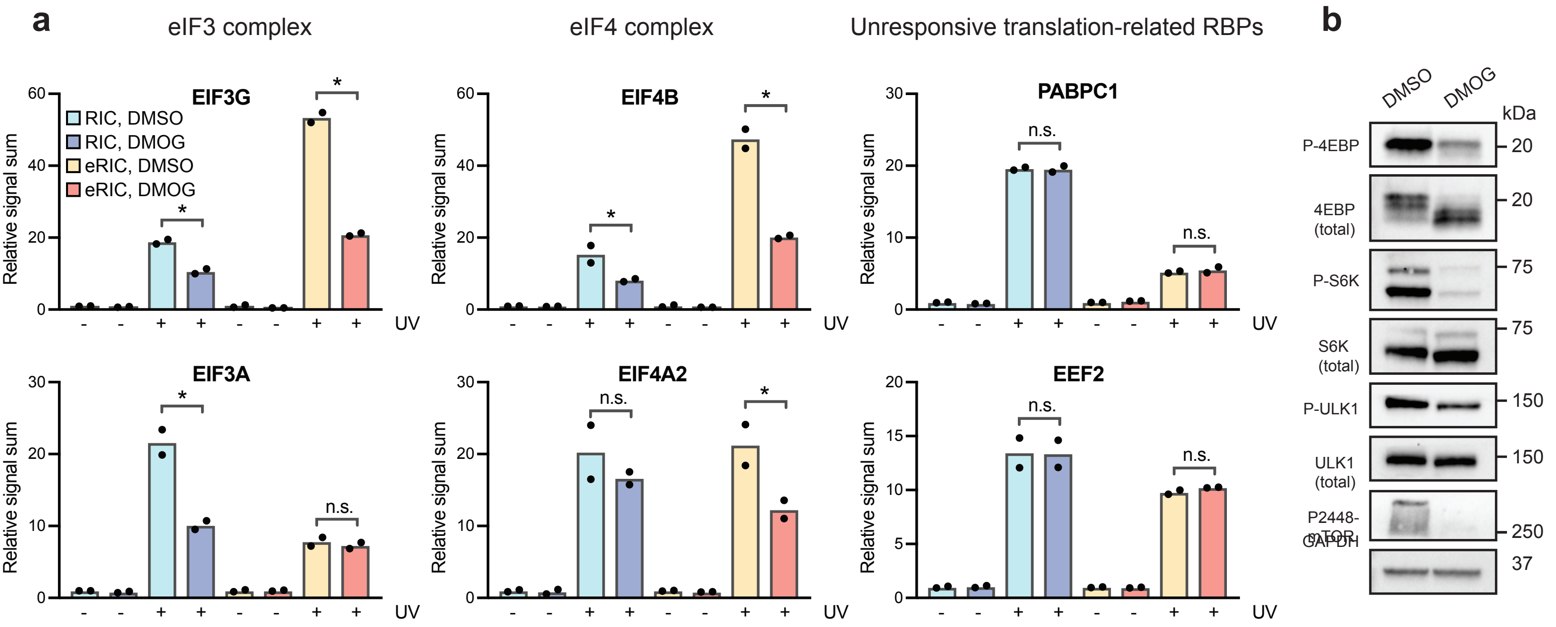

Supplementary Figure 2: eRIC reveals reduced binding of eIF3 and eIF4 to mRNA and activation of 4EBP upon DMOG treatment. (**a**) Normalized signal sum in eRIC and RIC samples of representative hits involved in mRNA translation initiation. Subunits of the eIF3 (left) and eIF4 complexes (middle) that respond to DMOG are shown in addition to unresponsive proteins (right). Note the different responses of some eIF3 and eIF4 subunits recovered by either of the two methods. -UV: non-crosslinked controls; +UV: crosslinked samples. eRIC and RIC values are expressed relative to the respective untreated control (-UV, DMSO). Data are shown as mean from two biologically independent experiments. * indicates FDR < 0.05 (moderated *t*-test). (**b**) DMOG treatment inhibits TOR. Western blot analysis for protein and phosphorylation abundance of the mTOR targets 4EBP, S6K and ULK1 and for phosphorylation levels of mTOR in cells incubated with 0.5mM DMOG or DMSO (vehicle) for 6 h. GAPDH was used as a loading control.

Protein	Description	log2(DMOG/DMSO)	FDR	Edupuganti *et al.*	Arguello *et al.*
TSN	translin	1.045	2.02E-14	-	-
TNPO1	transportin 1	0.870	2.43E-10	-	-
ARPP21	cAMP regulated phosphoprotein 21kDa	0.847	2.02E-14	-	-
CHERP	calcium homeostasis endoplasmic reticulum protein	0.709	1.60E-04	-	-
RBM12	RNA binding motif protein 12	0.619	2.03E-08	-	-
CPSF6	cleavage and polyadenylation specific factor 6	0.590	2.98E-09	Reader	Repelled
THRAP3	thyroid hormone receptor associated protein 3	0.491	1.59E-07	-	-
PRMT1	protein arginine methyltransferase 1	0.450	2.92E-03	-	-
R3HDM2	R3H domain containing 2	0.397	3.78E-06	-	-
SRSF1	serine/arginine-rich splicing factor 1	0.357	2.23E-02	Repelled	Repelled
TRA2A	transformer 2 alpha homolog (Drosophila)	0.351	2.22E-02	-	-
TRIM25	tripartite motif containing 25	0.339	1.37E-02	-	-
TACO1	translational activator of mitochondrially encoded cytochrome c oxidase I	0.339	3.34E-05	-	-
ILF2	interleukin enhancer binding factor 2	0.317	2.15E-03	-	Repelled
YTHDF3	YTH N(6)-methyladenosine RNA binding protein 3	0.308	1.32E-02	Reader	Reader
SRSF7	serine/arginine-rich splicing factor 7	0.308	4.29E-02	-	Reader
SRSF4	serine/arginine-rich splicing factor 4	0.306	4.42E-02	Repelled	Repelled
PCBP1	poly(rC) binding protein 1	0.304	3.98E-02	-	-
PUF60	poly(U) binding splicing factor 60KDa	0.263	4.18E-02	-	Reader/repelled
SF3B2	splicing factor 3b subunit 2	0.227	2.37E-02	-	Repelled
USP10	ubiquitin specific peptidase 10	-0.221	4.02E-02	Repelled	Repelled
EZR	ezrin	-0.240	3.52E-02	-	-
PUM1	pumilio RNA binding family member 1	-0.266	4.52E-03	-	-
EIF4A3	eukaryotic translation initiation factor 4A3	-0.267	2.37E-02	-	-
RACK1	receptor for activated C kinase 1	-0.294	1.30E-02	-	-
G3BP1	G3BP stress granule assembly factor 1	-0.295	2.32E-03	Repelled	Repelled
FXR1	FMR1 autosomal homolog 1	-0.311	5.91E-04	Reader	-
TRNAU1AP	tRNA selenocysteine 1 associated protein 1	-0.343	3.82E-05	-	-
LRPPRC	leucine rich pentatricopeptide repeat containing	-0.356	4.43E-05	-	Reader
FXR2	FMR1 autosomal homolog 2	-0.365	1.51E-03	Reader	-
G3BP2	G3BP stress granule assembly factor 2	-0.366	2.15E-02	Repelled	Repelled
CAPRIN1	cell cycle associated protein 1	-0.369	1.55E-04	Repelled	Repelled
BZW1	basic leucine zipper and W2 domains 1	-0.371	5.40E-05	-	-
DDX39B	DEAD-box helicase 39B	-0.372	6.91E-04	-	-
PPIL4	peptidylprolyl isomerase like 4	-0.376	6.02E-03	-	-
TBRG4	transforming growth factor beta regulator 4	-0.381	1.07E-04	-	-
SPTBN1	spectrin beta, non-erythrocytic 1	-0.391	3.02E-02	-	-
UPF1	UPF1 regulator of nonsense transcripts homolog (yeast)	-0.413	8.54E-06	-	-
BZW2	basic leucine zipper and W2 domains 2	-0.414	2.58E-02	-	-
EIF4G2	eukaryotic translation initiation factor 4 gamma 2	-0.415	5.62E-04	-	-
SARNP	SAP domain containing ribonucleoprotein	-0.456	3.87E-04	-	-
MRPL1	mitochondrial ribosomal protein L1	-0.467	6.33E-04	-	-
EIF3D	eukaryotic translation initiation factor 3 subunit D	-0.488	1.30E-04	-	-
PPIE	peptidylprolyl isomerase E	-0.497	2.24E-05	-	-
RPL30	ribosomal protein L30	-0.521	1.01E-03	-	Repelled
SUPT6H	SPT6 homolog, histone chaperone	-0.532	4.19E-04	-	-
SERBP1	SERPINE1 mRNA binding protein 1	-0.547	8.77E-05	-	-
HDLBP	high density lipoprotein binding protein	-0.615	4.79E-11	-	Repelled
MRPL39	mitochondrial ribosomal protein L39	-0.647	4.53E-02	-	-
SLIRP	SRA stem-loop interacting RNA binding protein	-0.651	9.27E-08	-	-
ETF1	eukaryotic translation termination factor 1	-0.658	9.06E-11	-	-
PUS1	pseudouridylate synthase 1	-0.699	2.83E-08	-	-
DDX3X	DEAD-box helicase 3, X-linked	-0.713	3.28E-12	-	-
FASTKD2	FAST kinase domains 2	-0.759	2.90E-10	-	-
EIF4A2	eukaryotic translation initiation factor 4A2	-0.784	8.44E-11	-	-
PNPT1	polyribonucleotide nucleotidyltransferase 1	-0.839	3.97E-09	-	-
EIF4A1	eukaryotic translation initiation factor 4A1	-1.073	2.02E-14	-	Repelled
EIF4H	eukaryotic translation initiation factor 4H	-1.220	2.02E-14	-	-
EIF4B	eukaryotic translation initiation factor 4B	-1.231	2.02E-14	-	-
EIF3G	eukaryotic translation initiation factor 3 subunit G	-1.356	2.02E-14	-	-
GRSF1	G-rich RNA sequence binding factor 1	-1.482	2.02E-14	-	-

Supplementary Table 1: DMOG-responsive RBPs identified by eRIC. Log2-fold change (FC) in DMOG- vs DMSO (vehicle)-treated and irradiated samples of the responsive proteins detected by eRIC (FDR < 0.05 (moderated t-test) and consistent FC of at least 10% in each replicate). Data correspond to two biologically independent experiments. The terms "Reader" and "Repelled" refer to the type of interaction with m6A the proteins were shown to display in the indicated studies.

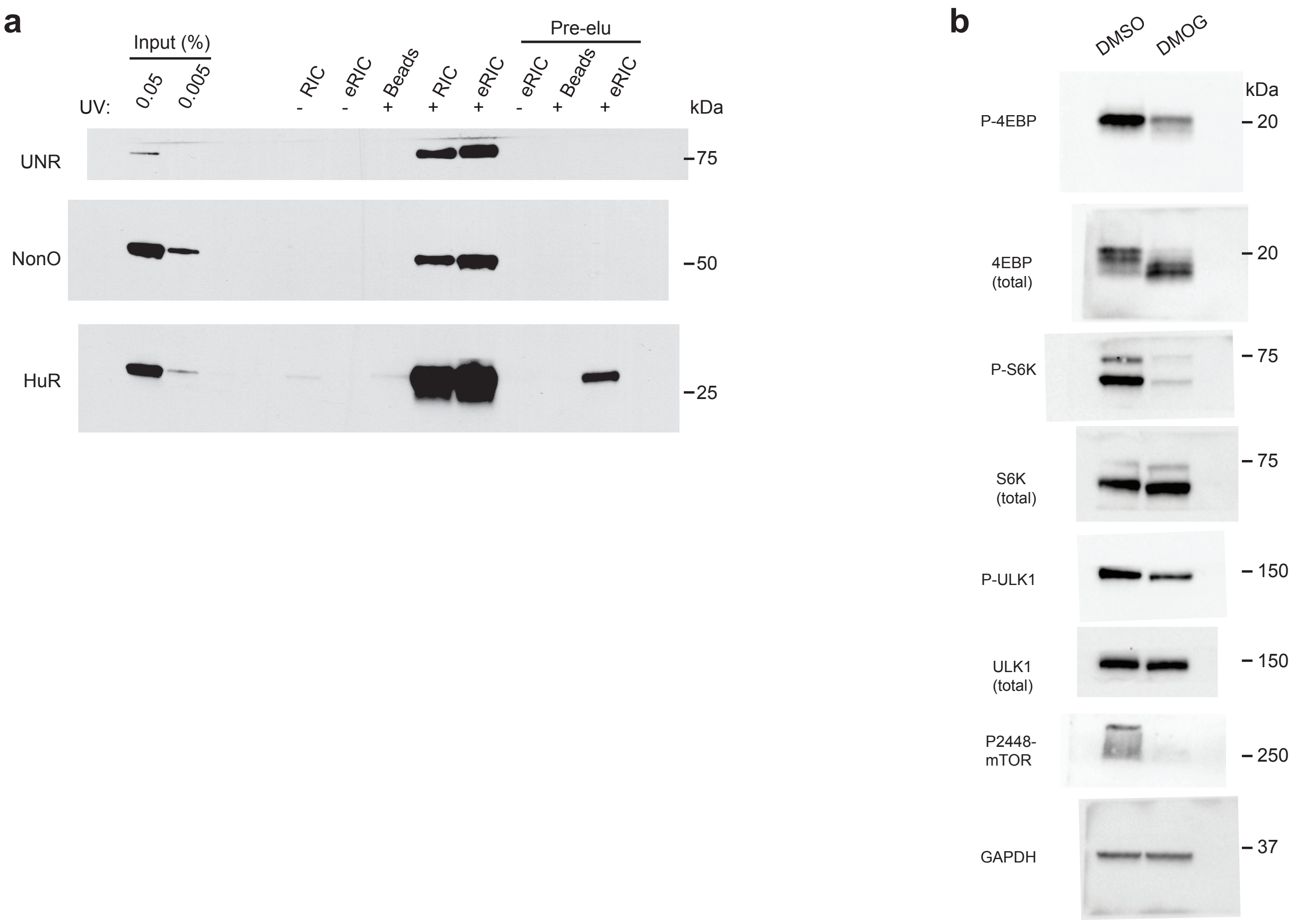

Supplementary Figure 3: Uncropped scans. Uncropped scans related to Fig. 1e (**a**) and Supplementary Fig. 2b (**b**).
