# Supplementary material for: Discovery of RNA-binding proteins and characterization of their dynamic responses by enhanced RNA interactome capture
Source: Nat Commun. 2018 Oct 23;9:4408. doi: 10.1038/s41467-018-06557-8 (PMC6199288; doi:10.1038/s41467-018-06557-8)
Supplement: Supplementary file 7 — Description of Additional Supplementary Files [file 41467_2018_6557_MOESM7_ESM.docx]

**Title:** Supplementary Data 1.
**Description:** Results of eRIC and RIC of Jurkat cells.

**Title:** Supplementary Data 2.
**Description:** Results of comparative eRIC and RIC of Jurkat cells exposed to 0.5mM DMOG or vehicle (DMSO) for 6 hours.

**Title:** Supplementary Data 3.
**Description:** Full list of GO terms enriched among RBPs differentially captured by eRIC and RIC. Clustering position refers to Fig.3.

**Title:** Supplementary Data 4.
**Description:** Full list of GO terms enriched among the DMOG-responsive RBPs identified by eRIC and/or RIC. BP: biological processes; CC: cellular componen
